# Phonon-Induced Geometric Chirality

**DOI:** 10.1021/acsnano.4c05978

**Published:** 2024-10-18

**Authors:** Carl P. Romao, Dominik M. Juraschek

**Affiliations:** † Department of Materials, ETH Zurich, CH-8093 Zurich, Switzerland; ‡ School of Physics and Astronomy, Tel Aviv University, Tel Aviv 6997801, Israel

**Keywords:** chiral phonons, chirality, nonlinear phononics, ultrafast dynamics, quantum materials

## Abstract

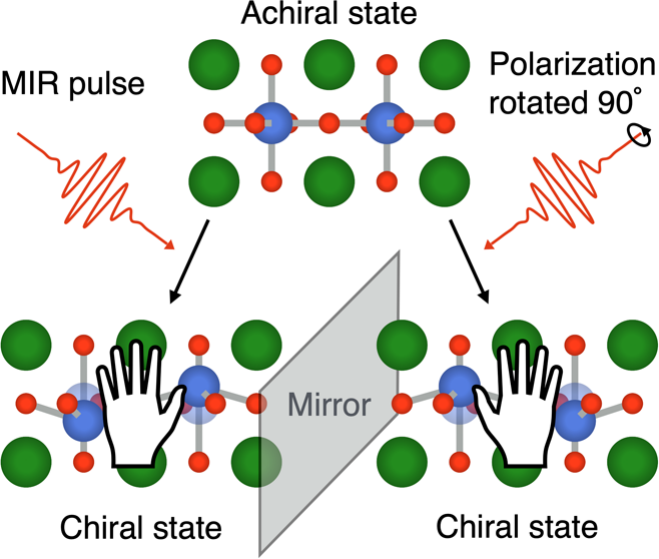

Chiral properties have seen increasing use in recent years, leading to the emerging fields of chiral quantum optics, plasmonics, and phononics. While these fields have achieved manipulation of the chirality of light and lattice vibrations, controlling the chirality of materials on demand has yet remained elusive. Here, we demonstrate that linearly polarized phonons can be used to induce geometric chirality in achiral crystals when excited with an ultrashort laser pulse. We show that nonlinear phonon coupling quasistatically displaces the crystal structure along phonon modes that reduce the symmetry of the lattice to that of a chiral point group corresponding to a chiral crystal. By reorienting the polarization of the laser pulse, the two enantiomers can be induced selectively. Therefore, *geometric chiral phonons* enable the light-induced creation of chiral crystal structures and therefore the engineering of chiral electronic states and optical properties.

## Introduction

Chirality describes a fundamental asymmetry of geometric objects and plays a central role in chemistry, biology, and physics. A geometric object is chiral when its mirror image cannot be superposed on the original system by any combination of rotations and translations. The chiral geometric arrangement of atoms in molecules and solids determines chemical reactivity and electronic phases, and is one of the central requirements for the formation of life.^1,2^ Geometric, or often called static, chirality can be extended to time-dependent systems, where a sense of handedness is applied to moving and rotating objects.^3^ The interplay of geometric and motion-based chirality can, for example, be observed in the chirality-induced spin selectivity (CISS) effect, which has been hypothesized to have influenced the origin of biological geometric homochirality by inducing enantioselective crystallization of RNA,^4^ and has recently been connected to chiral phonons.^5^ Chiral phonons represent a case of motion-based, or often called dynamic, chirality, as they conventionally describe circularly polarized lattice vibrations that carry linear and angular momentum and can therefore be assigned a well-defined helicity.^6−9^ This angular momentum of chiral phonons has in recent years been shown to lead to a plethora of emergent phenomena, including phonon Hall,^10−12^ phonon Zeeman,^13−16^ and phonon Einstein-de Haas effects,^17−19^ as well as equilibrium and ultrafast phono-magnetism.^13,14,20−33^ An intriguing question therefore arises of whether not just motion-based, but also geometric chirality can be induced with lattice vibrations, thereby creating a path to dynamically inducing electronic and optical properties that are connected to the chiral structure of materials.^34−36^

**Figure 1:**
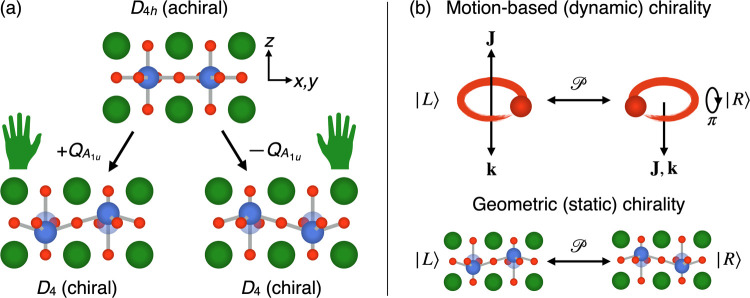
Phonon-induced geometric chirality. (a) Example of an *AB*O_3_ perovskite in the achiral point group *D*_4*h*_. The *A*_1*u*_ mode at *q* = 0 moves the two *B*-site cations in opposite directions along the tetragonal axis, *z*, breaking improper rotation symmetry and reducing the symmetry of the system to the chiral point group *D*_4_. Positive and negative displacements, ±*Q*_*A*_1*u*__, lead to two enantiomers. (b) In conventional chiral phonons, the circular motion of the atoms produces an angular momentum, **J**, which combined with the propagation direction of the phonon, **k**, breaks improper rotation symmetry.^8,9^ This is called motion-based chirality.^3^ In geometric chiral phonons, in contrast, the instantaneous displacement of the atoms itself, not the motion, breaks improper rotation symmetry, leading to geometric chirality. In both cases, the two enantiomeric states, |*L*⟩ and |*R*⟩, are connected by the parity operation, .

Here, we demonstrate theoretically and computationally that achiral crystals can be made chiral through the nonlinear excitation of phonon modes by intense ultrashort laser pulses. We perform a group-theoretical analysis to identify phonon modes whose displacement pattern breaks improper rotation symmetry, including inversion and mirror, and therefore leads to a chiral atomic structure (Figure 1a). We show that the crystal lattice can be quasistatically distorted along the eigenvectors of these phonon modes through the mechanism of nonlinear phononic rectification, which offsets the average of the atomic vibrations from their respective equilibrium positions, corresponding to a nonzero time-averaged mean value ⟨*Q*_*c*_⟩, where *Q*_*c*_ is the chiral phonon amplitude. The direction of displacement can be controlled through the polarization of the laser pulse, making chirality switching between the two enantiomers possible. We quantify the degree of induced chirality by introducing an electro-chiral susceptibility and we evaluate the chiral response to the excitation by an ultrashort pulse for the example of lithium triborate (LiB_3_O_5_).

## Results and Discussion

### Geometric Chiral Phonons

We begin with a group-theoretical analysis of phonon modes that are able to induce chiral crystal geometries. Chiral crystals do not contain any improper rotation symmetry, including inversion, mirrors, and rotoreflections. They are characterized by the 65 Sohncke space groups, which in turn are part of the 11 chiral point groups, shown in Supporting Table S1.^37^ In achiral crystals, there may exist phonon modes whose displacement pattern at an instant in time reduces the symmetry of the lattice to one of the Sohncke groups, therefore inducing a chiral crystal geometry. These phonons are to be contrasted with the circularly polarized chiral phonons commonly found in literature,^8,9^ where the motion of the atoms, not the instantaneous displacement itself, breaks improper rotation symmetry and leads to chirality, as illustrated in Figure 1b. We will therefore refer to these phonon modes as *geometric chiral phonons* in the remainder of the manuscript. We will focus on phonon modes at the center of the Brillouin zone (long-wavelength limit), which can be excited with light, and we further consider only systems for which the phonon energies are much smaller than the band gap so that any electronic transitions can be excluded. In our analysis, we utilize the character and multiplication tables of the Bilbao Crystallographic Server,^38^ and extract the group–subgroup relations corresponding to the phonon displacements using the ISOSUBGROUP software tool.^39^

In Supporting Tables S2 and S3, we show a list of the 32 point groups and indicate whether they possess inversion () or mirror () symmetries. The 11 chiral point groups possess neither inversion nor mirror symmetries. Point group *S*_4_(4̅) is a special case, which does not contain an inversion center or mirror planes, but contains a rotoreflection symmetry and is therefore not chiral. In the tables, we list the irreducible representations of phonon modes that break all inversion and mirror symmetries, as well as the respective subgroup symmetries they induce. Nearly all of them lead to chiral point groups and can therefore be considered geometric chiral phonons. Exceptions are the *B*_*u*_ modes of the *C*_4*h*_(4/m) point group, as well as the *A*_2_ modes of the *D*_2*d*_(4̅2m) point group, which break the inversion symmetry and all mirror symmetries, but which reduce the crystal symmetry to *S*_4_(4̅). For doubly and triply degenerate phonon modes, the induced subgroup depends on the chosen basis of the eigenvectors and we list all possible outcomes in the tables.

### Time-Averaged Geometric Chirality

We next examine how geometric chiral phonons can be excited by an ultrashort laser pulse to yield a macroscopic chiral crystal geometry. Harmonic excitations of geometric chiral phonons would leave the time-averaged crystal structure in its achiral state, where the atoms vibrate uniformly around their respective equilibrium positions. We therefore utilize nonlinear phononic rectification^40,41^ as a means to unidirectionally distort the lattice along the eigenvectors of a geometric chiral phonon mode. Nonlinear phononic rectification is a process wherein two phonon modes (denoted here *a* and *b*) that are driven coherently by a laser pulse couple to a third phonon mode (here the geometric chiral phonon mode *c*) through a three-phonon coupling of the type *Q*_*a*_*Q*_*b*_*Q*_*c*_. The geometric chiral phonon mode, *Q*_*c*_, therefore experiences a driving force proportional to the product of the driven phonon amplitudes, *Q*_*a*_*Q*_*b*_. Although the mean values of the driven phonon modes, ⟨*Q*_*a*_⟩ and ⟨*Q*_*b*_⟩, are zero, that of the driving force, ⟨*Q*_*a*_*Q*_*b*_⟩, is not. Hence, the mean of the geometric chiral phonon mode acquires a nonzero value following the mean-square amplitude of the driven phonons
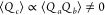
1Physically, this corresponds to a quasistatic displacement of the atoms along the eigenvectors of the geometric chiral phonon mode. As a result, a chiral crystal geometry can be induced. Positive and negative displacements along *Q*_*c*_ are related by mirror symmetry and therefore correspond to two enantiomers.

In Supporting Tables S2 and S3, we list all symmetry-allowed three-phonon couplings that contain the geometric chiral phonon. In order for a coupling to be symmetry-allowed, it needs to contain the fully symmetric representation.^40,41^ For nondegenerate phonon modes, *Q*_*a*_ and *Q*_*b*_ can correspond to the same (*a* = *b*) or to different irreducible representations (*a* ≠ *b*). For doubly and triply degenerate phonon modes, they generally correspond to the two orthogonal components of the same irreducible representation, e.g., *Q*_*a*_ ≡ *Q*_*a*(1)_ and *Q*_*b*_ ≡ *Q*_*a*(2)_. In order to achieve rectification with nondegenerate phonon modes, the eigenfrequencies of the two modes, Ω_*a*_ and Ω_*b*_, should be close together, so that the dephasing time of the two components is longer than the respective phonon lifetimes, κ_*a*_ and κ_*b*_, 2π(Ω_*a*_ – Ω_*b*_)^−1^ > κ_*a*/*b*_^–1^.

Achieving a measurable rectification of the crystal structure, ⟨*Q*_*c*_⟩ ≠ 0, relies on excitation of the driven phonon modes, *Q*_*a*_ and *Q*_*b*_, with large amplitudes, which can generally be achieved with resonant IR absorption, but not with impulsive stimulated Raman scattering. We therefore list the irreducible representations corresponding to the IR-active phonon modes in Supporting Tables S2 and S3. In centrosymmetric materials, geometric chiral phonons break inversion symmetry and can only couple to one infrared (IR)- and one Raman-active phonon mode at a time. In noncentrosymmetric materials, inversion symmetry is already broken, which lifts the principle of mutual exclusion for IR and Raman activity and allows geometric chiral phonons to couple to two IR-active phonon modes. Therefore, we expect the effect of phonon-induced geometric chirality to be most easily achievable in noncentrosymmetric materials and we will focus our analysis on these cases.

In Table 1, we show the 10 noncentrosymmetric-achiral point groups, in which three-phonon couplings of geometric chiral phonons to IR-active modes are possible. We further list example materials for each of the point groups in which these couplings can be found. We have limited our analysis to group–subgroup relations of point groups, as the emergence of chirality depends only on the point group. The corresponding relations for space groups can be determined using the ISODISTORT and ISOSUBGROUP software packages where necessary.^39^ An analysis of the group–subgroup relations of space groups yields that the displacement induced by geometric chiral phonons at the Brillouin-zone center always leads to one of the 43 nonenantiomorphic Sohncke groups and not to one of the 11 enantiomorphic space-group pairs. Pairs of enantiomers in the nonenantiomorphic Sohncke groups have no unambiguous “left” or “right” handedness defined by their symmetry operations. These pairs therefore represent a case of nonhanded chirality.^42,43^

**Table 1:** Geometric Chiral Phonons In Noncentrosymmetric-Achiral Point Groups^a^

point group	GCP	subgroup	IR-active modes	coupling	example materials
*C*_*s*_(m)	*A*″	*C*_1_(1)	*A*′(*x*, *y*), *A*″(*z*)	*A*″*A*″*A*′	KH_2_PO_4_, BaGa_4_Se_7_
*C*_2*v*_(mm2)	*A*_2_	*C*_2_(2)	*B*_1_(*x*), *B*_2_(*y*)	*A*_2_*B*_1_*B*_2_	LiB_3_O_5_, CdTiO_3_,
					GaFeO_3_
*S*_4_(4̅)	*B*	*C*_2_(2)	^*i*^*E*(*x*, *y*)^c^	*B*^*i*^*E*^*i*^*E^c^*	BPO_4_, Na_2_ZnSnS_4_
	^*i*^*E^c^*	*C*_1_(1)	^*i*^*E*(*x*, *y*), *B*(*z*)^c^	^*i*^*E*^*i*^*EB*^c^	
*C*_4*v*_(4mm)	*A*_2_	*C*_4_(4)	*E*(*x*, *y*)	*A*_2_*EE*	SrBaNb_2_O_6_
	*E*	*C*_1_(1), *C*_*s*_(m)^b^	*E*(*x*, *y*), *A*_1_(*z*)	*EEA*_1_	
*D*_2*d*_(4̅2m)	*A*_2_	*S*_4_(4̅)^b^	*E*(*x*, *y*)	*A*_2_*EE*	ZnGeP_2_, AgGaS_2_
	*B*_1_	*D*_2_(222)	*E*(*x*, *y*)	*B*_1_*EE*	
	*E*	*C*_1_(1), *C*_2_(2), *C*_*s*_(m)^b^	*E*(*x*, *y*), *B*_2_(*z*)	*EEB*_2_	
*C*_3*v*_(3m)	*A*_2_	*C*_3_(3)	*E*(*x*, *y*)	*A*_2_*EE*	LiNbO_3_, BiFeO_3_
	*E*	*C*_1_(1), *C*_*s*_(m)^b^	*E*(*x*, *y*), *A*_1_(*z*)	*EEA*_1_, *EEE*	
*C*_3*h*_(6̅)	^*i*^*E*″^c^	*C*_1_(1)	^*i*^*E*′(*x*, *y*), *A*″(*z*)^c^	^*i*^*E*″^*j*^*E*′*A*″^c^	LiCdBO_3_,
					BaZnBO_3_F
*C*_6*v*_(6mm)	*A*_2_	*C*_6_(6)	*E*_1_(*x*, *y*)	*A*_2_*E*_1_*E*_1_	GaBO_3_,
	*E*_1_	*C*_1_(1), *C*_*s*_(m)^b^	*E*_1_(*x*, *y*), *A*_1_(*z*)	*E*_1_*E*_1_*A*_1_	Ba_3_YbB_3_O_9_
	*E*_2_	*C*_2_(2), *C*_2*v*_(mm2)^b^	*E*_1_(*x*, *y*)	*E*_2_*E*_1_*E*_1_	
*D*_3*h*_(6̅m2)	*E*″	*C*_1_(1), *C*_2_(2), *C*_*s*_(m)^b^	*E*′(*x*, *y*), *A*_2_^″^(*z*)	*E*″*E*′*A*_2_^″^	Na_3_La_9_B_8_O_27_
*T*_*d*_(4̅3m)	*E*	*D*_2_(222), *D*_2*d*_(4̅2m)^b^	*T*_2_(*x*, *y*,*z*)	*ET*_2_*T*_2_	CsNbMoO_6_,
	*T*_1_	*P*_1_(1), *C*_*s*_(m),^b^*S*_4_(4̅),^b^	*T*_2_(*x*, *y*,*z*)	*T*_1_*T*_2_*T*_2_	Zn_4_B_6_O_13_
		*C*_3_(3)			

a We show the 10 noncentrosymmetric-achiral point groups, in which geometric chiral phonons can couple to two IR-active phonon modes, which makes their excitation through nonlinear phononic rectification feasible. For each point group, we list the irreducible representations of the geometric chiral phonons (GCP), the chiral subgroups that their displacement leads to, as well as the IR-active phonon modes they couple to and their coupling terms. We further show example materials for each of the cases, specifically, example materials which can be produced as optical single crystals. For doubly and triply degenerate modes, the induced subgroup depends on the chosen basis of eigenvectors, which may also lead to achiral subgroups.

b Subgroup is not chiral.

c *i*, *j* = 1, 2.

### Nonlinear Phonon Dynamics

The coherent phonon dynamics following the excitation by an ultrashort terahertz or mid-IR pulse can be captured in a semiclassical oscillator model, for which the input parameters can be computed from first principles.^41,44,45^ This has in the past been shown to yield quantitatively accurate estimates of the phonon amplitudes for semiconductors and insulators, where the photon energy of the laser pulse is far below the band gap.^46,47^ A minimal model of the phonon potential energy, which describes the nonlinear phonon dynamics, includes the harmonic terms for each phonon mode and the three-phonon coupling responsible for rectification:
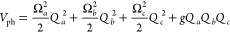
2where Ω_α_ is the eigenfrequency of phonon mode α, and *g* is the nonlinear phonon coupling coefficient. The phonon amplitudes, *Q*_α_, are given in units of Å√*u*, where *u* is the atomic mass unit. The light-matter coupling of the laser pulse to the IR-active phonons can be described as *V*_l-m_ = −**p**_α_ · **E**(*t*) = −*Z*_α,*i*_*Q*_α_*E*_*i*_(*t*), where **p**_α_ = **Z**_α_*Q*_α_ is the electric dipole moment of phonon mode α and **E**(*t*) is the electric field component of the laser pulse.  is the mode effective charge vector, where *Z*_n_^*^ is the Born effective charge tensor of atom *n*, **q**_α,*n*_ is its unitless phonon eigenvector, and *M*_*n*_ is its atomic mass. The electric field component of the laser pulse can be expressed as **E**(*t*) = (*E*(*t*) sin(ϕ), *E*(*t*) cos(ϕ), 0), where ϕ is an angle defining the orientation of linear polarization. , where *E*_0_ is the peak electric field, τ is the full width at half maximum of the pulse duration, and ω_0_ is the center frequency. We use the Einstein sum convention for the spatial index *i* ∈ {*x*, *y*, *z*}. The equations of motion can be written as
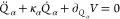
3where κ_α_ are the phonon linewidths arising from conventional phonon decay channels and *V* = *V*_ph_ + *V*_l-m_. The solutions of eq 3 can be approximated to first and second order in the electric field for the IR-active and geometric chiral phonon modes, respectively,^26^ see Methods.

### Electro-Chiral Susceptibility

Chirality can be quantified through various distance-based measures that compare an achiral parent structure with a corresponding symmetry-broken chiral structure. Here, we apply the continuous chirality measure,^37,48^ which is defined as
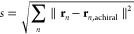
4where **r**_*n*,achiral_ is the position of atom *n* in the unit cell of the achiral parent structure and **r**_*n*_ is a position variable that varies continuously between the achiral and chiral structures. The index *n* runs over all atoms in the unit cell. The continuous chirality measure can be normalized as *S* = *s*/*N* with *N* = (∑_*n*_ ∥**r**_*n*,chiral_ – **r**_*n*,achiral_∥^2^)^1/2^, where **r**_*n*,chiral_ is the position of atom *n* in the chiral structure. In our analysis, **r**_*n*,achiral_ corresponds to the positions of the atoms in the equilibrium structure, whereas **r**_*n*_ corresponds to the time-dependent atomic coordinates along the eigenvectors of the geometric chiral phonon mode. The norm of the atomic displacements from the equilibrium structure is then simply given by . Generally, one cannot assume that an achiral material also has a corresponding stable chiral phase (or vice versa), which makes **r**_*n*,chiral_ undefined. In order to still define a measure, we select **r**_*n*,chiral_ as the positions of the atoms corresponding to the displace from the equilibrium structure with unit eigenvectors, .

With these considerations, we can express a time-dependent and mode-resolved normalized continuous chirality measure that is simply proportional to the unitless amplitude norm of the geometric chiral phonon, *S*_*c*_(*t*) = ∥*Q*_*c*_(*t*)∥/(Å√*u*). The conventional definition of the continuous chirality measure in eq 4 only returns positive values and therefore does not capture transitions between enantiomers. Positive and negative displacements along *Q*_*c*_ correspond to two enantiomers, however, and we need a way to distinguish them. We therefore drop the norm in our following definition of the continuous chirality measure, which then simply reads
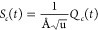
5We can now insert the solutions for the phonon amplitudes from eq 3 (see Methods) into the Fourier transform of eq 5, which yields a frequency-dependent continuous chirality measure,
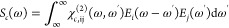
6
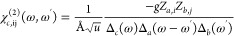
7

where Δ_α_(ω) = Ω_α_^2^ – ω^2^ + *iωκ*_α_, and *Z*_*a*,*i*_ and *Z*_*b*,*j*_ are the mode effective charge components of the IR-active phonon modes. χ_*c*,*ij*_^(2)^(ω, ω′) can be seen as a mode-resolved electro-chiral susceptibility that is a measure for the frequency-resolved response of the chiral displacements to the excitation by the laser pulse. In conventional nonlinear optics notation, the rectified component of the continuous chirality measure reads
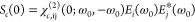
8The magnitude of the zero-frequency electro-chiral susceptibility can hence be used as a proxy for nonlinear phononic rectification and therefore phonon-induced chirality. The details of the dynamics depend on the parameters in eq 7 and on the form of the electric field components. If multiple geometric chiral phonon modes are involved in the dynamics, eq 4 contains a sum over them, see Methods.

### Phonon-Induced Geometric Chirality in Lithium Triborate

We now evaluate the theory developed in the previous sections for the example of LiB_3_O_5_, an ionic insulator that is well-suited for phonon driving. LiB_3_O_5_ crystallizes in the *Pna*2_1_ space group (point group *C*_2*v*_) and the 36-atom unit cell hosts 108 phonon modes with irreducible representations *A*_1_, *A*_2_, *B*_1_, and *B*_2_. The *A*_2_ modes in the system break all mirror symmetries and reduce the space group of the material to *P*2_1_ (point group *C*_2_), which is one of the Sohncke groups. Nonlinear phononic rectification along the eigenvectors of an *A*_2_ mode therefore creates a chiral crystal geometry. According to our analysis in Table 1, the *A*_2_ modes couple to the IR-active *B*_1_ and *B*_2_ modes, because the product of their irreducible representations contains the fully symmetric representation, *A*_1_ ⊂ *A*_2_ ⊗ *B*_1_ ⊗ *B*_2_. The *B*_1_ and *B*_2_ modes can be driven coherently by an ultrashort laser pulse. We perform density functional theory calculations to obtain the phonon eigenfrequencies, eigenvectors, and nonlinear couplings. (See Methods for the computational details.)

We investigate resonant driving of the high-frequency transverse optical (TO) *B*_1_ mode at Ω_*a*_/(2π) = 44.5 THz and *B*_2_ mode at Ω_*b*_/(2π) = 44.0 THz. We use a mid-IR pulse with a peak electric field of *E*_0_ = 15 MV/cm, pulse duration of τ = 100 fs, and center frequency of ω_0_/(2π) = 44.3 THz. The pulse is polarized ϕ = 45° in the *xy*-plane of the orthorhombic crystal to drive both IR-active modes simultaneously, which couple to all 27 *A*_2_ modes in the system. The *B*_1_ and *B*_2_ modes also couple to the fully symmetric *A*_1_ modes, which leave the crystal symmetry unchanged and are therefore not considered here. We pick the illustrative examples of the lowest and highest frequency *A*_2_ modes at 3.5 and 44.3 THz to demonstrate phonon-induced geometric chirality. The computed mode effective charges are *Z*_*a*,*x*_ = −1.1 *e*/√*u* and *Z*_*b*,*y*_ = −1.27 *e*/√*u*, where *e* is the elementary charge. The *A*_2_ modes are not IR-active, *Z*_*c*,*i*_ = 0. The computed coupling coefficients are *g* = 2 meV/(Å√*u*)^3^ for the *A*_2_(3.5) mode and *g* = 56 meV/(Å√*u*)^3^ for the *A*_2_(44.3) mode. We further assume phenomenological phonon linewidths of κ_α_ = 0.1Ω_α_/(2π).^13,44^

**Figure 2:**
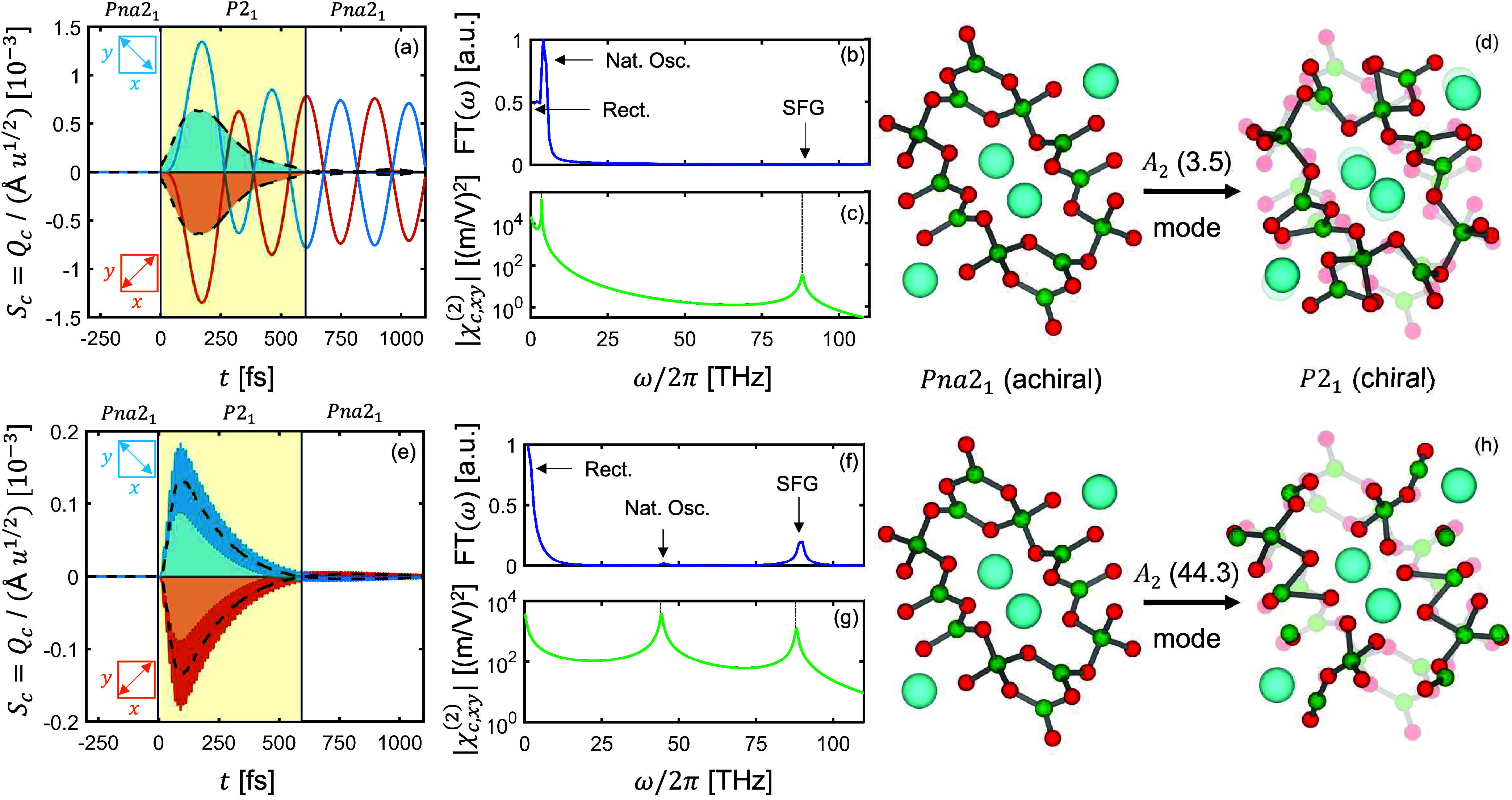
Phonon-induced geometric chirality in LiB_3_O_5_. Nonlinear excitation of the geometric chiral *A*_2_ modes at 3.5 (a-d) and 44.3 THz (e-h) following resonant driving of the IR-active *B*_1_(44.5) and *B*_2_(44.0) modes. (a,e) Time evolution of the *A*_2_ amplitudes, *Q*_*c*_, (blue and orange lines) and mean continuous chirality measures, ⟨*S*_*c*_⟩, (black dotted lines). Reorientation of the pulse polarization by 90° (insets) switches the signs of *Q*_*c*_ and ⟨*S*_*c*_⟩, selectively creating the two enantiomers. Shaded areas are a guide to the eye. (b,f) Fourier transforms of the *A*_2_ amplitudes, revealing rectified, natural-oscillation, and sum-frequency components. These features are captured by the electro-chiral susceptibility, |χ_*c,xy*_^(2)^(ω, ω_0_)|, shown in (c,g). (d,h) Structural distortions induced by the *A*_2_ modes, viewed along the *z*-axis of the crystal. Li ions are shown in blue, O in red, and B in green. The *A*_2_ displacements break the glide planes of the *Pna*2_1_ space group, which include mirror symmetries perpendicular to the direction of view, rendering the structure chiral.

We show the response of the system to excitation by the ultrashort mid-IR pulse as described by eqs 3 for the *A*_2_(3.5) mode in Figures 2(a–d) and for the *A*_2_(44.3) mode in Figures 2(e–h). We plot the phonon amplitudes, *Q*_*c*_, as well as the mean mode-resolved continuous chirality measures, ⟨*S*_*c*_⟩, in Figures 2(a,e). Before the laser pulse hits, *t* < 0, the crystal is in its achiral equilibrium characterized by space group *Pna*2_1_. After the action of the pulse, *t* > 0, the lattice is distorted impulsively along the eigenvectors of the *A*_2_ modes in response to the coupling to the *B*_1_ and *B*_2_ modes. The phonon amplitudes show oscillations that are offset from their equilibrium position at *Q*_*c*_ = 0. From the mean chirality measure ⟨*S*_*c*_⟩, we see that the symmetry of the crystal structure is reduced to *P*2_1_, a chiral state. ⟨*S*_*c*_⟩ decays quadratically with the lifetime of the IR-active phonon modes, and after *t* = 600 fs, only the oscillatory parts of the *A*_2_ modes remain, which means that on average, the crystal is back in its achiral *Pna*2_1_ state. When the polarization of the pulse is rotated by 90° in the *xy*-plane of the crystal, the sign of the driving force *Q*_*a*_*Q*_*b*_ changes, and accordingly so do those of *Q*_*c*_ and ⟨*S*_*c*_⟩. Because positive and negative displacements of the geometric chiral phonons are related by mirror symmetry, each of the enantiomers can be created selectively. Both *A*_2_ modes show net rectification, arising from the average force of the IR-active modes, ⟨*Q*_*a*_*Q*_*b*_⟩, because the dephasing time of the *B*_1_ and *B*_2_ modes is longer than their lifetimes. In addition, the two *A*_2_ modes show very different oscillatory behavior.

To disentangle the different features of the phonon dynamics, we show the Fourier transforms of the phonon amplitudes in Figure 2(b,f). There is a rectified component near zero frequency, arising from the difference frequency of the *B*_1_ and *B*_2_ modes at (Ω_*a*_ – Ω_*b*_)/(2π) = 0.5 THz, which corresponds to the quasistatic distortion. The *A*_2_(3.5) mode shows a clear peak at its eigenfrequency, because it is close to the difference-frequency component of the *Q*_*a*_*Q*_*b*_ driving force, which provides enough spectral weight for the *A*_2_(3.5) mode to be driven resonantly. The *A*_2_(44.3) mode in turn is far away from the frequency components of the *Q*_*a*_*Q*_*b*_ driving force and hence experiences only marginal spectral weight at its eigenfrequency. Its biggest contribution come from rectification, as well as from the sum-frequency component of the driving force at (Ω_*a*_ + Ω_*b*_)/(2π) = 88.5 THz, corresponding to sum-frequency generation. All three features are captured by the electro-chiral susceptibility, χ_*c*,*xy*_^(2)^, which we show in Figures 2(c,g) for resonant driving conditions, ω′ = ω_0_. The peaks near zero frequency correspond to rectification, the peaks at the eigenfrequencies of the *A*_2_ modes correspond to their natural oscillations, and the peaks at the sum frequency of the *B*_1_ and *B*_2_ modes correspond to sum-frequency generation, respectively.^26^

Finally, we show the structural distortions corresponding to the two *A*_2_ modes in Figures 2(d,h). The low-frequency *A*_2_(3.5) mode corresponds to a breathing mode of the borate framework, whereas the high-frequency *A*_2_(44.3) mode involves stretching and bending of B–O bonds. Both are transiently frozen in by the rectification mechanism. In an experiment, the total structural distortion would be a superposition of all 27 *A*_2_ modes of the system, showing varying components of the features displayed by the *A*_2_(3.5) and *A*_2_(44.3) modes, and with the chirality measure described by the total electro-chiral susceptibility (see Methods). In all calculations, we have evaluated the stability of the crystal with respect to the Lindemann criterion, predicting possible melting beyond a mean-square amplitude that displaces the atoms by more than 10% of the interatomic distance. The largest displacements along the coordinates of the IR-active phonons in our simulations is 1%, well within the stability criterion and experimental achievability.

## Conclusions

We have shown that geometric chiral phonon modes break improper rotation symmetry, reducing the symmetry of the crystal structure from an achiral to a chiral point group. These phonon modes can be unidirectionally displaced through nonlinear phononic rectification in response to the excitation by an ultrashort laser pulse, leading to a transient, phonon-induced chiral state. Reorienting the polarization of the laser pulse inverts the direction of the displacement, enabling enantioselective generation of chirality. In our example of LiB_3_O_5_, the rectified *A*_2_ modes reduce the crystal symmetry from space group *Pna*2_1_ (point group *C*_2*v*_) to *P*2_1_ (*C*_2_) and switching between the two enantiomers can be achieved by rotating the polarization of the pulse by 90° in the *xy*-plane of the orthorhombic crystal. The mechanism is generally applicable to materials in all 10 noncentrosymmetric-achiral point groups listed in Table 1.

Ultrafast pump–probe experiments have proven to be able to measure chirality in molecules.^49,50^ A possible experiment measuring phonon-induced geometric chirality in solids could involve time-resolved X-ray or electron diffraction following a mid-IR pump, which have proven to be powerful tools to resolve phonon dynamics.^19,51,52^ In LiB_3_O_5_, the reduction in symmetry would lead to the appearance of Bragg peaks in diffraction patterns at 0*k*l: *k* + *l* = 2*n* + 1, *h*0*l*: *h* = 2*n* + 1, *h*00: *h* = 2*n* + 1, and 0*k*0: *k* = 2*n* + 1, where *n* is any integer.^53^ A second possible experiment could involve X-ray chiral dichroism,^54,55^ in which the two enantiomers accessed by a reorientation of the pulse polarization could be distinguished. A third possible experiment involves the changes of gyrotropic optical properties that follow the creation of chirality. Changing the point group from *C*_2*v*_ to *C*_2_ in LiB_3_O_5_ introduces diagonal components to the gyrotropic tensor,^56^ which can be measured in a terahertz pump-optical probe setup. During the review process of this paper, an experiment using precisely this type of measurement on BPO_4_, which we list as a suitable candidate in Table 1, has surfaced,^57^ as well as theory proposing inducing chirality with strain.^58^ Other promising candidates for this type of measurement involve compounds of the *C*_3*v*_ point group (e.g., LiNbO_3_), in which a rectification of the *A*_2_ modes reduces the symmetry to *C*_3_, introducing diagonal gyrotropic tensor components. Furthermore, it is possible to induce gyrotropy in nongyrotropic materials, for example by rectifying the *E* modes in point group *T*_*d*_ (nongyrotropic), which reduces the symmetry to *D*_2_ (gyrotropic). A central challenge for future classifications of phonon-induced geometric chirality concerns finding an order parameter for chirality that is connected to physical observables, beyond geometric measures based on the geometric chiral phonon amplitude as used in this work.

Our prediction of phonon-induced geometric chirality and enantioselective switching introduces a paradigm in the optical control of solids, by enabling the engineering of chiral optical properties on demand and potentially allowing to induce chiral electronic phases, including chiral topology and superconductivity. The mechanism of inducing geometric chirality in solids furthermore constitutes a path to controlling magnetoelectric order (e.g., ferrotoroidal and ferroaxial) and chiral magnetism.^59−61^

## Methods

### Computational Details

We computed the phonon frequencies and eigenvectors at the Brillouin-zone center, as well as the Born effective charges using density functional perturbation theory (DFPT) as implemented in the *ab initio* software package Abinit.^62^ We used the Perdew–Burke–Ernzerhof (PBE) exchange–correlation functional with the dispersion correction of Grimme.^63^ For the plane-wave basis set, we used an energy cutoff of 38 Ha together with norm-conserving pseudopotentials obtained from the Abinit library, and a 6 × 6 × 6 Monkhorst-Pack grid to sample the Brillouin zone. A structural relaxation was performed prior to the DFPT calculations to an internal pressure of −10 kPa, yielding lattice parameters *a* = 8.37 Å, *b* = 7.35 Å, and *c* = 5.22 Å, consistent with experimental data.^64^ We used the eigenvectors obtained with DFPT to compute the lattice energy of the system as a function of the displacements of the *B*_1_, *B*_2_, and *A*_2_ modes on a 9 × 9 × 9 grid covering ±2 Å√*u*. We extracted the coupling constant *g* from a sixth-order polynomial fit to this grid using the polyfitn toolbox in MATLAB.

### Solutions of the Equations of Motion

The equations of motion for the phonon amplitudes are given by
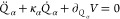
where κ_α_ are the phonon linewidths, α ∈ {*a*, *b*, *c*}, and *V* = *V*_*ph*_ + *V*_*l*-*m*_ from the main text. In detail, they read
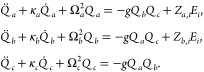
The solutions of these equations of motion in frequency space can be approximated to first and second order in the electric field for the IR-active and geometric chiral phonon modes, respectively.^26^ They yield
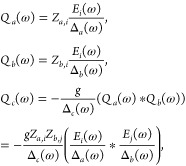
where  is the convolution operator and Δ_α_(ω) = Ω_α_^2^ – ω^2^ + *iωκ*_α_.

The equations of motion were further solved numerically to obtain the time evolutions of the geometric chiral phonon amplitude, *Q*_*c*_(*t*), and the continuous chirality measure, *S*_*c*_(*t*), in Figure 2a,e. The mean of the induced chirality, ⟨*S*_*c*_⟩, is shown there as dashed black lines and orange and blue shaded areas, which correspond to moving averages of *S*_*c*_(*t*) with a 0.2 fs window size and a 0.1 fs offset for the *A*_2_(3.5) mode (Figure 2a) and with a 0.02 fs window size and 0.002 fs offset for the *A*_2_(44.3) mode (Figure 2e). These values and the width of the yellow shaded area in Figure 2a,e, were chosen as guides to the eye.

### Total Electro-Chiral Susceptibility

If multiple geometric chiral phonon modes, labeled *c*_*l*_, are involved in the nonlinear phonon dynamics, the continuous chirality measure
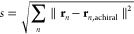
contains a sum over them, . Normalizing by the eigenvectors of all modes involved, we can then write the total electro-chiral susceptibility as
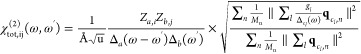
This expression reduces to the mode-resolved electro-chiral susceptibility in eq 7 of the main text when only one mode *c*_*l*_ ≡ *c* is considered.

## Supporting Information Available

Table of the 65 Sohncke groups and tables of geometric chiral phonons in all 32 point groups (PDF)

## Notes

**Preprint:** A preprint version of this article is available on the arXiv: Romao, C. P.; Juraschek, D. M. Phonon-induced geometric chirality. **2023**, 2311.00824, arXiv, https://arxiv.org/abs/2311.00824
