# Supplementary material for: Phonon-Induced Geometric Chirality
Source: ACS Nano. 2024 Oct 18;18(43):29550–7. doi: 10.1021/acsnano.4c05978 (PMC11526423; doi:10.1021/acsnano.4c05978)
Supplement: Supplementary file 1 — nn4c05978_si_001.pdf [file nn4c05978_si_001.pdf]

Supporting Information: Phonon-induced geometric chirality

Carl P. Romao,[1,*] and Dominik M. Juraschek[2,*]

[1]*Department of Materials, ETH Zurich, CH-8093 Zurich, Switzerland*
[2]*School of Physics and Astronomy, Tel Aviv University, Tel Aviv 6997801, Israel*

Point group	Space groups	Space group #
$C_1(1)$	P_1	1
$C_2(2)$	$P2$, $P2_1$, $C2$	3–5
$D_2(222)$	$P222$, $P222_1$, $P2_12_12$, $P2_12_12_1$, $C222_1$, $C222$, $F222$, $I222$, $I2_12_12_1$	16–24
$C_4(4)$	$P4$, $P4_1{}^*$, $P4_2$, $P4_3{}^*$, $I4$, $I4_1$	75–80
$D_4(422)$	$P422$, $P42_12$, $P4_122^*$, $P4_12_12^\dagger$, $P4_222$, $P4_22_12$, $P4_322^*$, $P4_32_12^\dagger$, $I422$, $I4_122$	89–98
$C_3(3)$	$P3$, $P3_1{}^*$, $P3_2{}^*$, $R3$	143–146
$D_3(32)$	$P312$, $P321$, $P3_112^*$, $P3_121^\dagger$, $P3_212^*$, $P3_221^\dagger$, $R32$	149–155
$C_6(6)$	$P6$, $P6_1{}^*$, $P6_5^*$, $P6_2{}^\dagger$, $P6_4{}^\dagger$, $P6_3$	168–173
$D_6(622)$	$P622$, $P6_122^*$, $P6_522^*$, $P6_222^\dagger$, $P6_422^\dagger$, $P6_322$	177–182
$T(23)$	$P23$, $F23$, $I23$, $P2_13$, $I2_13$	195–199
$O(432)$	$P4_32$, $P4_232$, $F432$, $F4_132$, $I432$, $P4_332^*$, $P4_132^*$, $I4_132$	207–214
*†Enantiomorphic space-group pairs		

TABLE S1. **Chiral point groups and Sohncke space groups.** The 11 chiral point groups host the 65 Sohncke groups that do not contain an improper rotation axis (including inversion, mirrors, and rotoreflections) and therefore describe chiral crystals. Of the Sohncke groups, 22 are enantiomorphic, meaning that a crystal structure and its enantiomorphic pair, which are interconverted by an odd number of mirror-symmetry operations, can be differentiated by the symmetry operations of their space group. For example, left-handed quartz crystallizes in space group $P3_221$ (no. 154) and right-handed quartz crystallizes in space group $P3_121$ (no. 152). In the other 43 Sohncke groups, the chirality of the crystal arises from the chirality of the asymmetric unit rather than that of the symmetry operations. Crystals in these space groups are nonhanded chiral, which means that pairs of enantiomers cannot unambiguously be assigned left- and right-handedness. This is also known as the Ruch shoe-potato problem [1].

Point group	$\mathcal{I}$	$\mathcal{M}$	Chiral mode	Subgroup	Nonlinear phonon coupling	IR-active modes
Triclinic						
$C_1(1)$	✗	✗				
$C_i(\bar{1})$	✓	✗	A_u	$C_1(1)$	$A_uA_uA_g$	$A_u(x,y,z)$
Monoclinic						
$C_2(2)$	✗	✗				
C_s(m)	✗	✓	A''	$C_1(1)$	$A''A''A'$	$A'(x,y)$, $A''(z)$
C_{2h}(2/m)	✓	✓	A_u	$C_2(2)$	$A_uA_uA_g$, $A_uB_uB_g$	$B_u(x,y)$, $A_u(z)$
Orthorhombic						
$D_2(222)$	✗	✗				
C_{2v}(mm2)	✗	✓	A_2	$C_2(2)$	$A_2A_2A_1$, $A_2B_1B_2$	$B_1(x)$, $B_2(y)$, $A_1(z)$
D_{2h}(mmm)	✓	✓	A_u	$D_2(222)$	$A_uA_uA_g$, $A_uB_{\alpha u}B_{\alpha g}$ $(\alpha=1,2,3)$	$B_{3u}(x)$, $B_{2u}(y)$, $B_{1u}(z)$
Tetragonal						
$C_4(4)$	✗	✗				
$S_4(\bar{4})$	✗	✗	B	$C_2(2)$	BBA, $B{}^iE{}^iE$ $(i=1,2)$	${}^iE(x,y)$, $B(z)$
			${}^iE$	$C_1(1)$	${}^iE{}^jEA$, ${}^iE{}^iEB$ $(i,j=1,2;\ i\neq j)$	
C_{4h}(4/m)	✓	✓	A_u	$C_4(4)$	$A_uA_uA_g$, $A_uB_uB_g$, $A_u{}^iE_u{}^jE_g$ $(i,j=1,2)$	${}^iE_u(x,y)$, $A_u(z)$
			B_u	$S_4(\bar{4})^*$	$B_uB_uA_g$, $B_uA_uB_g$, $B_u{}^iE_u{}^jE_g$ $(i,j=1,2;\ i\neq j)$	
$D_4(422)$	✗	✗				
C_{4v}(4mm)	✗	✓	A_2	$C_4(4)$	$A_2A_2A_1$, $A_2B_1B_2$, A_2EE	$E(x,y)$, $A_1(z)$
			E	$C_1(1)$, $C_s(\mathrm{m})^*$	EEA_α, EEB_α $(\alpha=1,2)$	
$D_{2d}(\bar{4}2\mathrm{m})$	✗	✓	A_2	$S_4(\bar{4})^*$	$A_2A_2A_1$, $A_2B_1B_2$, A_2EE	$E(x,y)$, $B_2(z)$
			B_1	$D_2(222)$	$B_1B_\alpha A_\alpha$, B_1EE	
			E	$C_1(1)$, $C_2(2)$, $C_s(\mathrm{m})^*$	EEA_α, EEB_α $(\alpha=1,2)$	
D_{4h}(4/mmm)	✓	✓	A_{1u}	$D_4(422)$	$A_{1u}A_{\alpha u}A_{\alpha g}$, $A_{1u}B_{\alpha u}B_{\alpha g}$, $A_{1u}E_uE_g$ $(\alpha=1,2)$	$E_u(x,y)$, $A_{2u}(z)$
Trigonal						
$C_3(3)$	✗	✗				
$C_{3i}(\bar{3})$	✓	✗	A_u	$C_3(3)$	$A_uA_uA_g$, $A_u{}^iE_u{}^iE_g$ $(i=1,2)$	${}^iE_u(x,y)$, A_u
			${}^iE_u$	$C_1(1)$	${}^iE_u{}^iE_uA_g$, ${}^iE_u{}^jE_uA_g$, ${}^iE_u{}^iE_gA_u$, ${}^iE_u{}^jE_gA_u$, ${}^iE_u{}^jE_u{}^iE_g$, ${}^iE_u{}^iE_u{}^jE_g$ $(i,j=1,2;\ i\neq j)$	
$D_3(32)$	✗	✗				
C_{3v}(3m)	✗	✓	A_2	$C_3(3)$	$A_2A_2A_1$, A_2EE	$E(x,y)$, $A_1(z)$
			E	$C_1(1)$, $C_s(\mathrm{m})^*$	EEA_α, EEE $(\alpha=1,2)$	
$D_{3d}(\bar{3}\mathrm{m})$	✓	✓	A_{1u}	$D_3(32)$	$A_{1u}A_{\alpha u}A_{\alpha g}$, $A_{1u}E_uE_g$ $(\alpha=1,2)$	$E_u(x,y)$, $A_{2u}(z)$
			E_u	$C_1(1)$, $C_2(2)$, $C_s(\mathrm{m})^*$	$E_uE_uA_{\alpha g}$, $E_uE_gA_{\alpha u}$, $E_uE_uE_g$ $(\alpha=1,2)$	

*Subgroup is not chiral

TABLE S2. **Geometric chiral phonons by point group I.** We show the point groups of the triclinic, monoclinic, orthorhombic, tetragonal, and trigonal crystal systems. We mark the presence (✓) or absence (✗) of inversion and mirror symmetries, the irreducible representations of the geometric chiral phonons, the subgroups they lead to upon displacement, the possible three-phonon couplings relevant to nonlinear phononic rectification, and the irreducible representations of the IR-active phonon modes. For doubly and triply degenerate modes, the induced subgroup depends on the chosen basis of eigenvectors, which may also lead to achiral subgroups. Point group $S_4(\bar{4})$ has no inversion or mirror symmetries, but still contains improper rotations, which makes it achiral.

Point group	$\mathcal{I}$	$\mathcal{M}$	Chiral mode	Subgroup	Nonlinear phonon coupling	IR-active modes
Hexagonal						
$C_6(6)$	✗	✗				
$C_{3h}(\bar{6})$	✗	✓	A''	$C_3(3)$	$A''A''A'$, $A''{}^iE'{}^jE''$ $(i,j=1,2)$	${}^iE'(x,y)$, $A''(z)$
			${}^iE''$	$C_1(1)$	${}^iE''{}^iE''A'$, ${}^iE''{}^jE''A'$, ${}^iE''{}^iE'A''$, ${}^iE''{}^jE'A''$, ${}^iE''{}^iE''{}^jE'$ $(i,j=1,2;\ i\neq j)$	
$C_{6h}(6/\mathrm{m})$	✓	✓	A_u	$C_6(6)$	$A_uA_uA_g$, $A_uB_uB_g$, $A_u{}^iE_{\alpha u}{}^iE_{\alpha g}$ $(i=1,2;\ \alpha=1,2)$	${}^iE_{1u}(x,y)$, $A_u(z)$
			${}^iE_{2u}$	$C_2(2)$	${}^iE_{2u}{}^jE_{2u}A_g$, ${}^iE_{2u}{}^jE_{2g}A_u$, ${}^iE_{2u}{}^jE_{1u}B_g$, ${}^iE_{2u}{}^jE_{1g}B_u$, ${}^iE_{2u}{}^iE_{\alpha u}{}^jE_{\alpha g}$, ${}^iE_{2u}{}^jE_{\alpha u}{}^iE_{\alpha g}$ $(i,j=1,2;\ \alpha=1,2)$	
$D_6(662)$	✗	✗				
$C_{6v}(6\mathrm{mm})$	✗	✓	A_2	$C_6(6)$	$A_2A_2A_1$, $A_2B_1B_2$, $A_2E_\alpha E_\alpha$ $(\alpha=1,2)$	$E_1(x,y)$, $A_1(z)$
			E_1	$C_1(1)$, $C_s(\mathrm{m})^*$	$E_1E_1A_\alpha$, $E_1E_2B_\alpha$, $E_1E_1E_2$ $(\alpha=1,2)$	
			E_2	$C_2(2)$, $C_{2v}(\mathrm{mm2})^*$	$E_2E_2A_\alpha$, $E_2E_1B_\alpha$, $E_2E_\alpha E_\alpha$ $(\alpha=1,2)$	
$D_{3h}(\bar{6}\mathrm{m2})$	✗	✓	A_1''	$D_3(32)$	$A_1''A_\alpha''A_\alpha'$, $A_1''E'E''$ $(\alpha=1,2)$	$E'(x,y)$, $A_2''(z)$
			E''	$C_1(1)$, $C_2(2)$, $C_s(\mathrm{m})^*$	$E''E''A_\alpha'$, $E''E'A_\alpha''$, $E''E''E'$ $(\alpha=1,2)$	
$D_{6h}(6/\mathrm{mmm})$	✓	✓	A_{1u}	$D_6(662)$	$A_{1u}A_{\alpha u}A_{\alpha g}$, $A_{1u}B_{\alpha u}B_{\alpha g}$, $A_{1u}E_{\alpha u}E_{\alpha g}$ $(\alpha=1,2)$	$E_{1u}(x,y)$, $A_{2u}(z)$
			E_{2u}	$C_2(2)$, $D_2(222)$, $C_{2v}(\mathrm{mm2})^*$	$E_{2u}E_{2u}A_{\alpha g}$, $E_{2u}E_{2g}A_{\alpha u}$, $E_{2u}E_{1u}B_{\alpha g}$, $E_{2u}E_{1g}B_{\alpha u}$, $E_{2u}E_{\alpha u}E_{\alpha g}$ $(\alpha=1,2)$	
Cubic						
$T(23)$	✗	✗				
$T_h(\mathrm{m}\bar{3})$	✓	✓	A_u	$T(23)$	$A_uA_uA_g$, $A_u{}^iE_u{}^iE_g$, $A_uT_uT_g$ $(i=1,2)$	$T_u(x,y,z)$
			${}^iE_u$	$D_2(222)$	${}^iE_u{}^jE_uA_g$, ${}^iE_u{}^jE_gA_u$, ${}^iE_u{}^iE_u{}^jE_g$, ${}^iE_u{}^jE_u{}^iE_g$, ${}^iE_uT_uT_g$ $(i,j=1,2)$	
$O(432)$	✗	✗				
$T_d(\bar{4}3\mathrm{m})$	✗	✓	A_2	$T(23)$	$A_2A_2A_1$, A_2EE, $A_2T_1T_2$	$T_2(x,y,z)$
			E	$D_2(222)$, $D_{2d}(\bar{4}2\mathrm{m})^*$	EEA_α, EEE, $ET_\alpha T_\beta$ $(\alpha,\beta=1,2)$	
			T_1	$P_1(1)$, $C_s(\mathrm{m})$,* $S_4(\bar{4})$,* $C_3(3)$	$T_1T_1A_1$, $T_1T_2A_2$, T_1T_1E, $T_1T_\alpha T_\beta$ $(\alpha,\beta=1,2)$	
$O_h(\mathrm{m}\bar{3}\mathrm{m})$	✓	✓	A_{1u}	$O(432)$	$A_{1u}A_{\alpha u}A_{\alpha g}$, $A_{1u}E_uE_g$, $A_{1u}T_{\alpha u}T_{\alpha g}$ $(\alpha=1,2)$	$T_{1u}(x,y,z)$
			E_u	$D_2(222)$, $D_4(422)$, $D_{2d}(\bar{4}2\mathrm{m})^*$	$E_uE_uA_{\alpha g}$, $E_uE_gA_{\alpha u}$, $E_uE_uE_g$, $E_uT_{\alpha u}T_{\beta g}$ $(\alpha,\beta=1,2)$	

*Subgroup is not chiral

TABLE S3. **Geometric chiral phonons by point group II.** We show the point groups of the hexagonal and cubic crystal systems. We mark the presence (✓) or absence (✗) of inversion and mirror symmetries, the irreducible representations of the geometric chiral phonons, the subgroups they lead to upon displacement, the possible three-phonon couplings relevant to nonlinear phononic rectification, and the irreducible representations of the IR-active phonon modes. For doubly and triply degenerate modes, the induced subgroup depends on the chosen basis of eigenvectors, which may also lead to achiral subgroups.

[1] R. B. King, “Chirality and handedness: The Ruch ‘shoe-potato’ dichotomy in the right-left classification problem,” Ann. N. Y. Acad. Sci. **988**, 158–170 (2003).
